# From symptoms to strategy: pre-procedural NYHA-class as a key to risk stratification and personalized TAVR-management

**DOI:** 10.3389/fcvm.2026.1638908

**Published:** 2026-04-17

**Authors:** Birgit Markus, Philipp Lauten, Georgios Chatzis, Leonard Steinheisser, Torben Zaun, Nikolaos Patsalis, Styliani Syntila, Harald Lapp, Bernhard Schieffer, Julian Kreutz

**Affiliations:** 1 Department of Medicine, Philipps University of Marburg, Marburg, Germany; 2 Department of Cardiology, Angiology, and Intensive Care Medicine, University Hospital of Marburg, Marburg, Germany; 3 Department of Cardiology, Central Hospital of Bad Berka, Bad Berka, Germany

**Keywords:** New York heart association (NYHA), outcome, personalized therapy, risk stratification, transcatheter aortic valve replacement (TAVR)

## Abstract

**Background:**

Transcatheter aortic valve replacement (TAVR) has become a widely used treatment option for severe aortic stenosis (AS), particularly in elderly and multimorbid patients. The New York Heart Association (NYHA) classification, which assesses the severity of heart failure (HF), is a key factor influencing TAVR outcomes. However, its impact on procedural success, complications, and outcomes remains underrepresented in recent studies.

**Methods:**

In this multicenter study, data from 2,256 patients who underwent TAVR between 2017 and 2022 at two high-volume German Heart Centers were analyzed. Demographics, comorbidities, and peri-procedural parameters were evaluated to determine the influence of pre-procedural NYHA classification on complications, hospital stay, and outcomes. Multivariable logistic regression analyses were performed to assess the independent prognostic impact of pre-procedural NYHA class on 30-day and 1-year mortality.

**Results:**

NYHA class III/IV prior to the procedure was associated with higher peri-procedural complication rates, prolonged hospital stays, and increased mortality compared to class NYHA I/II. In particular, the rates for cardiopulmonary resuscitation (5.3% vs. 0.7%; *p* < 0.001), acute coronary intervention (1.9% vs. 0.0%; *p* = 0.006), vasopressor use >6 h (11.7% vs. 1.6%; *p* < 0.001), and renal replacement therapy (6.8% vs. 0.2%; *p* < 0.001) were higher. Procedure-related complications like vascular closure device failure (4.9% vs. 1.3%; *p* = 0.008), need for vascular surgery (9.0% vs. 6.3%; *p* = 0.002), and blood transfusion (9.4% vs. 4.7%; *p* = 0.017) were more common in NYHA IV. Median hospital stay was longer in NYHA IV (10.0 vs. 6.0 days; *p* < 0.001). The 30-day mortality rate was 8.3% (NYHA IV) vs. 1.4% (NYHA I/II), and 1-year mortality was 19.2% vs. 5.2% (*p* < 0.001). After multivariable adjustment for relevant clinical confounders, NYHA class IV remained independently associated with both 30-day and 1-year mortality.

**Conclusions:**

Pre-procedural NYHA class provides important prognostic information in patients undergoing TAVR, with higher symptom burden associated with increased peri-procedural risk and mortality. These findings highlight the relevance of comprehensive pre-procedural evaluation and optimized timing of intervention. Incorporating functional status into pre-procedural assessment may support risk stratification and individualized patient management.

## 1 Introduction

In recent years, transcatheter aortic valve replacement (TAVR) has become an increasingly established treatment option for aortic stenosis (AS). Although initially reserved for high-risk or inoperable patients, the procedure has rapidly evolved, driven by increasingly promising data since its introduction into clinical practice (1). Today, TAVR is broadly accepted and also utilized in patients with intermediate or low risk (2, 3). In line with demographic changes and updated european guidelines lowering the recommended age threshold for TAVR from >75 to ≥70 years in patients with tricuspid aortic stenosis and suitable anatomy (Class I, Level A), an increasing proportion of elderly and multimorbid patients are currently undergoing TAVR (4). Outcomes in this increasingly heterogeneous patient cohort are shaped by various factors, with pre-procedural clinical status remaining a major predictor (5–7).

Pathophysiological mechanisms triggered by AS are characterized by considerable complexity, resulting in varying severity of heart failure (HF) and left ventricular systolic dysfunction, which can be routinely assessed and quantified using the *New York Heart Association* (NYHA) classification. The development of HF in the context of untreated AS is a gradual process that typically evolves over several years before becoming clinically apparent. In contrast, the correction of AS via TAVR reduces left ventricular afterload, improves hemodynamics, promotes reverse remodeling, alleviates HF symptoms, and enhances both survival and quality of life (8–11). The timing of the TAVR procedure, therefore, appears to be a clinically relevant factor even in well-characterized patient subgroups (12–15). Major TAVR trials, including the PARTNER and CoreValve trials, as well as large registry data, such as TVT and FRANCE-2, consistently show significant improvements in patient outcomes in dependence on different NYHA classifications (2, 3, 16–19).

Although the association between pre-operative NYHA class and outcomes following cardiac surgery is well-established and plays a crucial role in therapeutic decision-making, its specific impact on TAVR success and early complication rates remains unclear. Importantly, NYHA functional class reflects the overall clinical status and degree of cardiac decompensation at the time of hospital admission and may result from multifactorial etiologies, including concomitant coronary artery disease or cardiomyopathies. Notably, this factor has not yet been fully integrated into the decision-making process for TAVR (20–22). To gain a deeper understanding, it is crucial to thoroughly investigate how pre-procedural cardiac decompensation, as reflected by the NYHA classification—independent of the underlying cause of symptoms-affects outcomes after TAVR. This investigation will be pivotal for optimizing patient selection and peri-procedural management in an aging population, ultimately improving both individual patient outcomes and the overall safety and effectiveness of the procedure. Moreover, as the aging population grows, healthcare systems face increasing economic pressures, underscoring the importance of efficient and personalized management strategies. The current study seeks to contribute to closing this knowledge gap in medical practice. By investigating the impact of the symptom-based pre-procedural NYHA classification on peri-interventional complications and outcomes, it aims to provide a more nuanced understanding of the predictive value of the pre-procedural NYHA classification in the context of TAVR.

## 2 Materials and methods

### 2.1 Study design

This retrospective, multicenter study analyzed data from patients who underwent transcatheter aortic valve replacement (TAVR) at two German heart centers: the University Hospital of Marburg and the Central Hospital of Bad Berka. The study period was from January 2017 to December 2022. Data were extracted from institutional electronic health records and procedural documentation systems, then collected using a predefined case report framework with harmonized variable definitions across both centers. Inclusion criteria were the performance of a TAVR procedure within the study period and documentation of the pre-procedural New York Heart Association (NYHA) functional class at hospital admission, which serves as an indicator of cardiac decompensation and the severity of heart failure. Patients with missing NYHA classification at admission were excluded. In cases involving multiple TAVR procedures during the study period, only the initial procedure was considered. Peri-procedural outcomes and clinical data from multiple domains, including physician reports, laboratory findings, admission electrocardiograms (ECGs), imaging studies, and peri-procedural documentation, were analyzed and compared across different NYHA classes. The NYHA functional class was assessed at hospital admission, prior to the TAVR procedure, by the treating clinical team. It was used as an integrative clinical marker of global cardiac decompensation. For analysis purposes, NYHA classes I and II were combined and compared with NYHA classes III and IV, respectively. Due to the retrospective study design, systematically attributing NYHA class to specific etiologies was not feasible. At both participating centers, coronary angiography was routinely performed in close temporal proximity to TAVR. In the presence of relevant coronary artery disease, percutaneous coronary intervention was performed according to institutional standards. Consequently, by the time of TAVR, patients were considered near-fully revascularized, with no remaining critical, high-grade coronary stenoses. The primary outcome of the study was all-cause mortality at 30 days and one year after TAVR. Secondary outcomes included peri-procedural complications such as cardiopulmonary resuscitation, acute coronary intervention due to peri-procedural myocardial infarction, the need for vasopressor therapy lasting over six hours, renal failure requiring renal replacement therapy, failure of the vascular closure device, the need for vascular surgery, the need for a blood transfusion, stroke, and length of in-hospital stay. Mortality follow-up was conducted at the predefined time points of 30 days and one year after the initial TAVR procedure. Mortality status was determined using institutional medical records and follow-up information documented at the participating centers.

### 2.2 Statistical analysis

Statistical analyses were performed using SPSS (version 29, IBM Corp., Armonk, NY) and GraphPad Prism (version 10.1, GraphPad Software, San Diego, CA). All statistical tests were two-sided, and a *p*-value of ≤0.05 was considered statistically significant. Categorical variables were presented as counts and percentages and compared using Pearson's chi-squared test. Fisher's exact test was applied when expected cell frequencies were low. Continuous variables are reported as the mean ± standard deviation or the median with the interquartile range, as appropriate. Normality was assessed using standard distributional criteria. Group comparisons were performed using one-way analysis of variance (ANOVA) with Welch's correction for unequal variances, or the Kruskal–Wallis test for non-normally distributed data. *post hoc* pairwise comparisons were conducted with the appropriate adjustments for multiple testing, including the Bonferroni correction to reduce type I error and the Games–Howell test in the presence of unequal variances. For adjusted analyses of categorical variables, the chi-squared *n* – 1 test was applied, when applicable. Multivariable logistic regression analyses were performed to evaluate the independent association between pre-procedural NYHA class and 30-day and 1-year mortality. Owing to the limited number of early events, a parsimonious model for 30-day mortality included age, chronic kidney disease (KDIGO stage ≥ G3a), and reduced left ventricular ejection fraction (LVEF ≤ 45%). For 1-year mortality, the model was expanded to additionally adjust for atrial fibrillation, diabetes mellitus, chronic obstructive pulmonary disease (COPD), coronary artery disease, and body mass index (BMI). NYHA class was entered as a categorical variable with NYHA I/II as the reference category. Sensitivity analyses incorporated the Society of Thoracic Surgeons (STS) score as a continuous variable. Odds ratios (ORs) with 95% confidence intervals (CIs) were reported, and model fit was assessed using the Hosmer–Lemeshow goodness-of-fit testing. All analyses were conducted on an available-case (complete-case) basis. No imputation of missing data was performed, and the number of patients with valid data for each variable is reported accordingly.

### 2.3 Ethics

The study was approved by the Ethics Committee of Philipps University of Marburg (reference RS 22/65, approved on October 27, 2022) and the Ethics Committee of the Medical Association of Thuringia (approved on July 13, 2023). Both approvals were obtained following the principles of the Declaration of Helsinki.

## 3 Results

Between January 2017 and December 2022, 2,256 patients underwent the TAVR procedure. NYHA classification status on admission was documented in 2,161 patients. The 30-day all-cause mortality rate of the entire NYHA cohort was 3.1%, and the 1-year all-cause mortality rate was 8.9%. Patients with NYHA IV were significantly older, with a mean age of 80.5 ± 6.3 years compared to those with NYHA I/II and III classification (I/II: 79.1 ± 5.9 years, III: 79.7 ± 6.4 years; *p* = 0.013). A comprehensive listing of demographics and comorbidities is provided in Table 1.

**Table 1:** Demographic data and comorbidities are stratified by NYHA classification at admission.

Demographics and comorbidities	*n*	NYHA I/II	NYHA III	NYHA IV	*p*-value
Number of patients	2,161	558	1,337	266	
Age (years)^b^	2,161	79.1 (±5.9)	79.7 (±6.4)	80.5 (±6.3)	**0**.**013**
Male sex^a^	2,161	319 (57.2)	699 (52.3)	137 (51.5)	0.120
BMI (kg/m^2^)^b^	2,154	27.9 (±4.9)	28.7 (±5.3)	28,6 (±5.6)	**0**.**006**
Cardiac bypass surgery^a^	2,161	34 (6.1)	84 (6.3)	16 (6.0)	0.978
Atrial fibrillation^a^	2,161	158 (28.3)	502 (37.5)	121 (45.5)	**<0**.**001**
CHD^a^	2,161	290 (52.0)	761 (56.9)	173 (65.0)	**0**.**002**
PAD ≥ stage 2^a^	2,160	41 (7.3)	210 (15.7)	39 (14.7)	**<0**.**001**
Arterial hypertension^a^	2,160	486 (87.1)	1,201 (89.9)	241 (90.6)	0.151
Hyperlipidemia^a^	2,159	390 (70.0)	909 (68.0)	180 (67.7)	0.666
Diabetes mellitus^a^	2,161	205 (36.8)	533 (39.9)	130 (48.9)	**0**.**004**
Insulin-dependent diabetes mellitus^a^	2,160	86 (15.4)	232 (17.4)	59 (22.2)	0.057
Nicotine abuse (>5py)^a^	2,161	81 (14.5)	218 (16.3)	40 (15.0)	0.592
Chronic renal failure KDIGO ≥ stage 3^a^	2,161	179 (32.1)	593 (44.4)	171 (64.3)	**<0**.**001**
Renal replacement therapy^a^	2,161	11 (2.0)	19 (1.4)	9 (3.4)	0.085
COPD ≥ GOLD 2^a^	2,160	37 (6.6)	166 (12.4)	40 (15.0)	**<0**.**001**
Cerebral stroke^a^	2,158	20 (3.6)	58 (4.3)	15 (5.6)	0.401
Malignant disease^a^	2,160	33 (5.9)	142 (10.6)	34 (12.8)	**0**.**001**
Pacemaker^a^	2,161	45 (8.1)	158 (11.8)	41 (15.4)	**0**.**005**

*n*, number of patients with valid data; BMI, body mass index; CHD. coronary heart disease; PAD, peripheral arterial disease; NYHA, New York Heart Association; py, pack years; KDIGO, Kidney Disease Improve Global Outcomes; COPD, chronic obstructive pulmonary disease.

Bold value indicates statistical significance.

a *n* (%).

b Mean (SD).

The subsequent analysis focused on pre-interventional parameters, including ECGs, chest x-rays, laboratory findings, and echocardiography, as summarized in Table 2. Notably, pre-interventional ECGs showed no significant differences between NYHA classes. However, chest x-rays revealed progressively worsening signs of pulmonary venous congestion, pleural effusions, and infiltrates with increasing NYHA stage. Laboratory analyses demonstrated progressively higher levels of C-reactive protein (CRP) and hemoglobin A1c (HbA1c), along with decreased hemoglobin and hematocrit levels, elevated leukocyte counts, and significantly higher N-terminal pro-B-type natriuretic peptide (NT-proBNP) concentrations correlating with advanced NYHA stages. Echocardiography showed a significantly higher prevalence of at least moderately reduced left ventricular ejection fraction (LVEF ≤ 45%) and advanced concomitant valvular heart disease (grade 2/3 mitral and/or tricuspid regurgitation) in patients with higher NYHA classifications. Moreover, the peak flow velocity across the aortic valve (Vmax) and mean aortic gradient (*Δ*Pmean) were found to decrease progressively with increasing NYHA stage, indicating more low flow and low gradient AS in advanced stages of HF (Table 2).

**Table 2:** Pre-interventional diagnostic examinations in different NYHA classes.

Pre-interventional diagnostic examinations	*n*	NYHA I/II	NYHA III	NYHA IV	*p*-value
Number of patients	2,161	558	1,337	266	
Pre-interventional ECG					
Heart rate (bpm)^4^	2,161	74.2 ± 14.1	75.6 ± 14.9	77.5 ± 16.6	0.103
Right bundle branch block^a^	2,161	53 (9.5)	97 (7.3)	14 (5.3)	0.076
Left bundle branch block^a^	2,161	48 (8.6)	112 (8.4)	28 (10.5)	0.522
First degree AV block^a^	2,161	62 (11.1)	126 (9.4)	25 (9.4)	0.514
Pre-interventional chest x-ray					
Pneumonia/ infiltrates	2,161	1 (0.2)	36 (2.7)	15 (5.6)	**<0**.**001**
Pleural effusion^a^	2,130	31 (5.6)	137 (10.2)	60 (22.6)	**<0**.**001**
Other congestion signs^a^	2,161	19 (3.4)	87 (6.5)	27 (10.2)	**<0**.**001**
Pre-interventional laboratory parameters					
CRP (mg/dL)^c^3	2,155	2.3 [1.0–5.1]	3.3 [1.4–8.7]	6.2 [2.0–15.4]	**<0**.**001**
HbA1c (%)^c^	1,739	5.9 (5.5–6.4)	6.0 (5.6–6.6)	6.1 (5.6–6.8)	**<0**.**001**
Hemoglobin (g/L)^c^	2,161	130 (119–140)	127 (114–137)	122 (109–130)	**<0**.**001**
Hematocrit (L/L)^c^	2,158	0.39 (0.36–0.41)	0.38 (0.35–0.41)	0.37 (0.34–0.40)	**<0**.**001**
Leukocyte count (g/L)^c^	2,151	7.5 (6.3–8.8)	7.7 (6.5–9.3)	8.2 (6.6–9.9)	**<0**.**001**
Platelet count (g/L)^c^	2,161	203 (169–245)	210 (171–258)	209 (164–262)	0.129
Sodium (mmol/L)^c^	2,143	141 (139–143)	140 (138–143)	141 (138–143)	**0**.**045**
NT proBNP (pg/mL)^c^	1,633	968 (482–2,309)	1,391 (464–3,332)	3,315 (1,397–8,477)	**<0**.**001**
Pre-interventional echocardiography					
^c^LVEF ≤45%^a^	2,139	93 (16.8)	317 (24.0)	99 (37.4)	**<0**.**001**
Vmax aortic valve (mmHg)^b^	1,630	4.3 (3.9–4.6)	4.1 (3.8–4.5)	4.0 (3.4–4.4)	**<0**.**001**
*Δ*Pmean (mmHg)^b^	1,929	47.0 (39.0–57.0)	46.0 (37.0–60.0)	43 (32.0–55.0)	**0**.**005**
Aortic Valve Area (cm^2^)^c^	2,024	0.75 (±0.17)	0.75 (±0.19)	0.73 (±0.19)	0.295
Concomitant vitium (grade 2/3) of the mitral valve^a^	2,133	151 (27.5)	384 (29.1)	118 (45.0)	**<0**.**001**
Concomitant vitium (grade 2/3) of the tricuspid valve^a^	2,134	94 (17.1)	270 (20.4)	70 (26.7)	**0**.**006**
Bicuspid aortic valve^a^	2,139	60 (10.8)	107 (8.1)	22 (8.3)	0.162

*n*, number of patients with valid data; bpm, beats per minute; AV Block, atrioventricular block; CRP, C-reactive protein; HbA1c, hemoglobin A1c, NT-proBNP, N-terminal prohormone of brain natriuretic peptide; LVEF, left ventricular ejection fraction; Vmax, peak transvalvular velocity of aortic valve; *Δ*Pmean, mean aortic valve pressure gradient.

Bold value indicates statistical significance.

a *n* (%).

b Mean (SD).

c Median [IQR].

**Figure 1:**
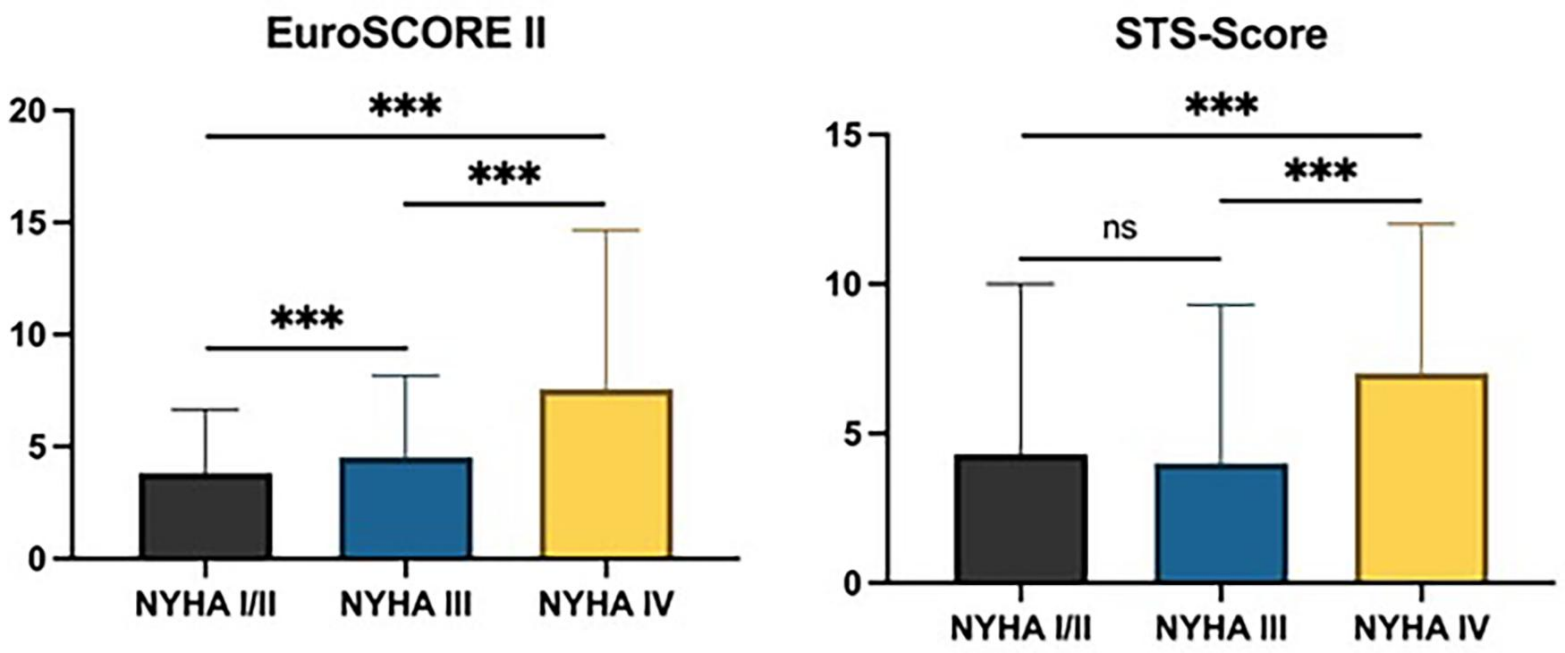
Pre-interventional documentation of EuroSCORE II and STS score in different NYHA classes. Ns, not significant; ***p < 0.001.

The median of the pre-interventional EuroSCORE II score was 3.8 (IQR 2.4–6.7) in NYHA I/II, 4.5 (IQR 2.8–8.2) in NYHA III, and 7.6 (IQR 4.6–14.7) in NYHA IV (*p* < 0.001). Similarly, the median *Society of Thoracic Surgeons* (STS) score was 4.3 (IQR 2.0–10.0) in NYHA I/II, 4.0 (IQR 2.5–9.3) in NYHA III, and 7.0 (IQR 3.8–12.0) in NYHA IV (*p* < 0.001) (Figure 1).

Multivariable logistic regression analyses were performed to evaluate the association between pre-procedural NYHA class and mortality after adjustment for baseline risk factors. Owing to the limited number of 30-day events, a parsimonious model including age, chronic kidney disease (KDIGO ≥ G3a), and reduced left ventricular ejection fraction (LVEF ≤ 45%) was applied. In this model, NYHA class IV was associated with increased 30-day mortality (OR 4.76, 95% CI 2.05–11.07, *p* < 0.001), whereas NYHA class III did not reach statistical significance (OR 1.71, 95% CI 0.79–3.73, *p* = 0.175). Increasing age (OR 1.048 per year, *p* = 0.036) and chronic kidney disease (OR 1.80, *p* = 0.030) were additional predictors of early mortality, while reduced LVEF was not significant after adjustment. Sensitivity analysis incorporating the composite STS score yielded consistent results (OR 5.79, *p* = 0.004).

For 1-year mortality, a fully adjusted model including age, chronic kidney disease, reduced LVEF, atrial fibrillation, diabetes mellitus, chronic obstructive pulmonary disease, coronary artery disease, and body mass index demonstrated a persistent association between NYHA class IV and outcome (OR 3.28, 95% CI 1.98–5.46, *p* < 0.001), whereas NYHA class III showed a borderline association (OR 1.50, 95% CI 0.97–2.32, *p* = 0.067). Additional predictors of long-term mortality included age (OR 1.042 per year, *p* = 0.004), chronic kidney disease (OR 1.72, *p* = 0.001), atrial fibrillation (OR 1.75, *p* < 0.001), COPD (OR 1.65, *p* = 0.019), and lower BMI (OR 0.96 per kg/m^2^, *p* = 0.020), while reduced LVEF was not associated with mortality after adjustment. In sensitivity analyses including only NYHA class and the STS score, both NYHA class III (OR 2.53, *p* = 0.004) and NYHA class IV (OR 5.48, *p* < 0.001) were associated with increased 1-year mortality, alongside the STS score itself (OR 1.045 per percentage point increase, *p* = 0.003).

To evaluate peri-interventional complications associated with TAVR, pre-procedural NYHA classifications were categorized, indicating a significant association between higher pre-procedural NYHA classes and increased peri-interventional complications. In particular, the incidence of peri-interventional cardiopulmonary resuscitation increased with higher NYHA class: 0.7% in NYHA I/II, 3.0% in NYHA III, and 5.3% in NYHA IV (*p* < 0.001). Similarly, the need for acute coronary intervention due to peri-interventional myocardial infarction was also correlated with higher NYHA classes (0.0% in NYHA I/II, 0.8% in NYHA III, and 1.9% in NYHA IV; *p* = 0.006). In addition, prolonged need for vasopressor therapy (>6 h post-procedure) to maintain a mean arterial pressure of about 65 mmHg was more common in patients with higher NYHA classes (1.6% in NYHA I/II, 5.5% in NYHA III, and 11.7% in NYHA IV; *p* < 0.001). The incidence of renal failure requiring temporary renal replacement therapy (RRT) was 0.2% in NYHA I/II, 2.1% in NYHA III, and 6.8% in NYHA IV (*p* < 0.001). Patients classified as NYHA IV experienced the highest rate of vascular closure device failure (4.9%), significantly higher than the 1.3% observed in NYHA I/II and 2.8% in NYHA III (*p* = 0.008). Additionally, NYHA IV patients required the highest frequency of vascular surgery, with rates of 6.3% in NYHA I/II, 4.5% in NYHA III, and 9.0% in NYHA IV (*p* = 0.002). Required blood transfusion increased with advanced NYHA classes (4.7% in NYHA I/II, 7.9% in NYHA III, and 9.4% in NYHA IV; *p* = 0.017) These findings are summarized in Table 3.

**Table 3:** Peri-procedural complications in different NYHA classes.

	*n*	NYHA I/II	NYHA III	NYHA IV	*p*-Wert
Number of patients	2,161	558	1,337	266	
Cardiopulmonary resuscitation^a^	2,160	4 (0.7)	30 (2.2)	14 (5.3)	**<0**.**001**
Aortic dissection^a^	2,161	1 (0.2)	5 (0.4)	0 (0.0)	0.853
Acute coronary intervention for periprocedural MI^a^	2,161	0 (0.0)	11 (0.8)	5 (1.9)	**0**.**006**
Switch to SAVR^a^	2,161	1 (0.2)	4 (0.3)	1 (0.4)	0.713
Need for vasopressor therapy (>6 h post intervention)^a^	2,160	9 (1.6)	74 (5.5)	31 (11.7)	**<0**.**001**
New onset atrial fibrillation^a^	2,161	12 (2.2)	41 (3.1)	7 (2.6)	0.520
Need for pacemaker implantation^a^	2,158	70 (12.6)	204 (15.3)	38 (14.3)	0.319
Pericardial tamponade^a^	2,161	12 (2.2)	32 (2.4)	10 (3.8)	0.363
Renal failure requiring RRT^a^	2,161	1 (0.2)	28 (2.1)	18 (6.8)	**<0**.**001**
Dissection of the femoral artery^a^	2,161	6 (1.1)	9 (0.7)	4 (1.5)	0.335
Failure of the vascular closure device^a^	2,161	7 (1.3)	37 (2.8)	13 (4.9)	**0**.**008**
Need for vascular surgery^a^	2,161	20 (6.3)	60 (4.5)	24 (9.0)	**0**.**002**
Requirement for blood transfusion within the first 48 h following TAVR^a^	2,161	26 (4.7)	105 (7.9)	25 (9.4)	**0**.**017**
Cerebral stroke^a^	2,161	13 (2.3)	27 (2.0)	8 (3.0)	0.779

MI, myocardial infarction; SAVR, surgical aortic valve replacement; RRT, renal replacement therapy.

a *n* (%).

**Figure 2:**
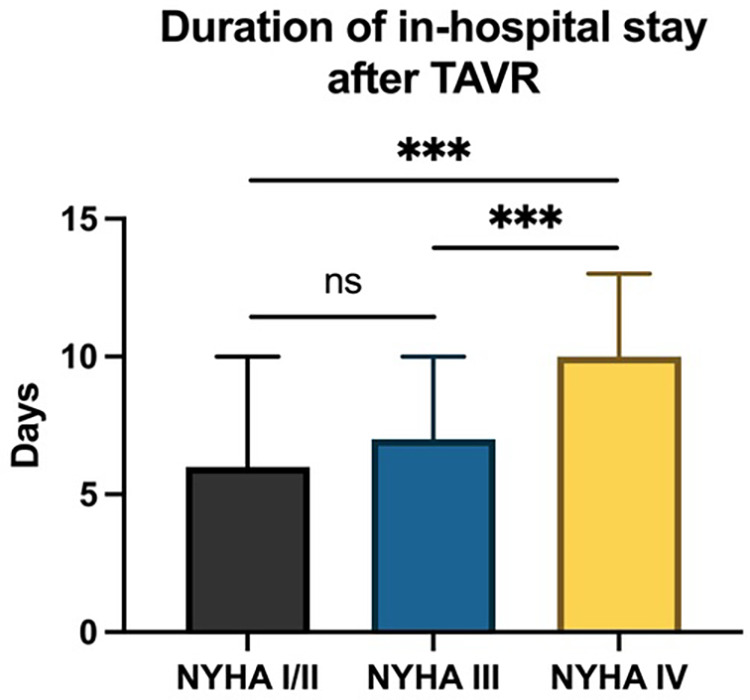
Duration of hospital stay following TAVR according to different NYHA classes. Ns, not significant; ***p < 0.001.

To evaluate post-procedural outcomes after TAVR, hospital length of stay and patient survival were analyzed according to NYHA classification. The median length of in-hospital stays following TAVR increased with higher NYHA functional classes (Figure 2). Specifically, patients classified as NYHA I/II had a median stay of 6.0 days (IQR 4.0–10.0), while NYHA III patients had a median inpatient stay of 7.0 days (IQR 5.0–10.0). NYHA IV patients remained in hospital for 10.0 days (IQR 6.0–13.0), indicating a statistically significant difference (*p* < 0.001).

**Figure 3:**
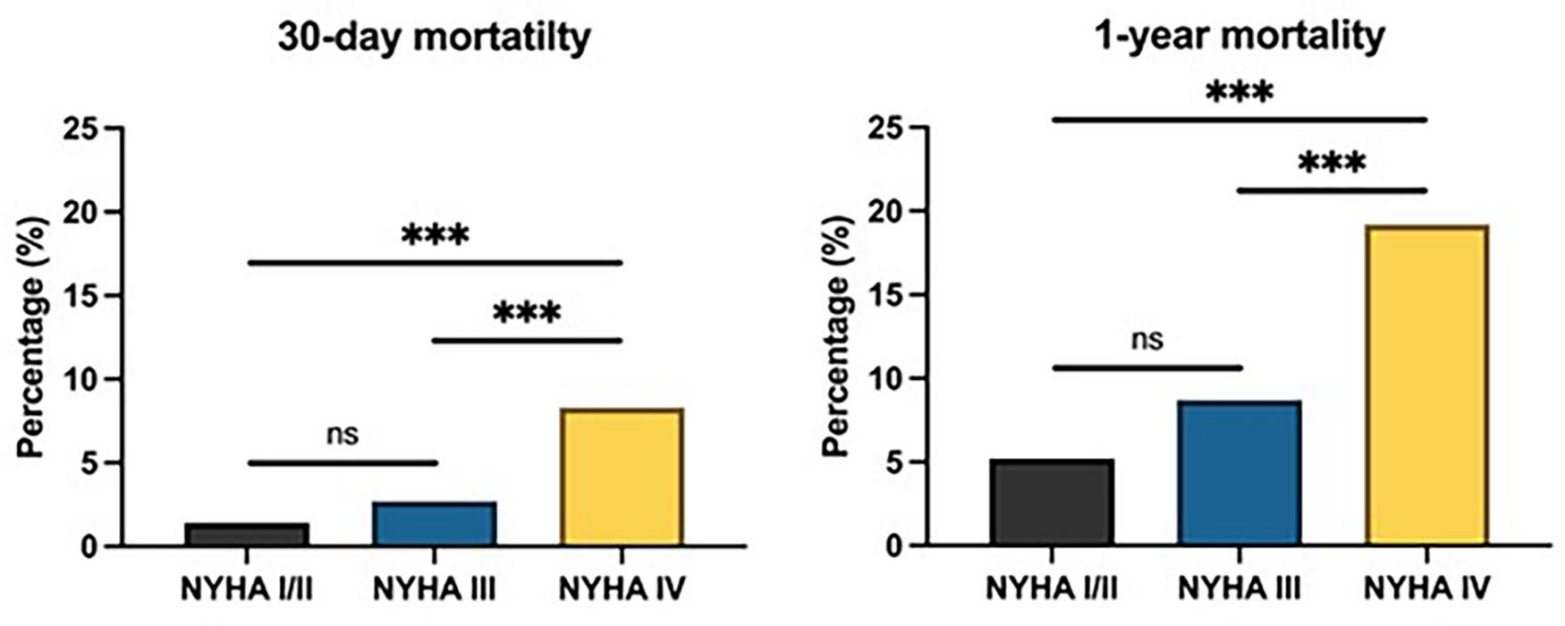
30-day and 1-year mortality following TAVR according to different NYHA classes. Ns, not significant, ***p < 0.001.

30-day mortality rates were higher with worsening NYHA class: 1.4% for NYHA I/II, 2.7% for NYHA III, and 8.3% for NYHA IV (*p* < 0.001). This trend was consistent with the patterns observed for peri-interventional complications and length of in-hospital stay. Similarly, 1-year mortality rates showed a significant increase with NYHA classification, with 5.2% for NYHA I/II, 8.7% for NYHA III, and 19.2% for NYHA IV (*p* < 0.001). These findings are summarized in Figure 3.

## 4 Discussion

In this study, pre-procedural NYHA classification emerged as an important prognostic marker of peri-procedural complications, length of hospital stays, and both short- and long-term mortality after TAVR. Importantly, the association between advanced NYHA class and mortality persisted after multivariable adjustment and remained consistent in STS-adjusted sensitivity analyses, suggesting that functional status provides prognostic information beyond established risk scores. These findings highlight the value of incorporating pre-procedural NYHA status into clinical decision-making. Furthermore, our data suggest that a comprehensive pre-interventional assessment integrating NYHA classification, comorbidities, and overall clinical condition supports individualized treatment strategies, particularly in high-risk TAVR patients, while emphasizing the need for earlier identification and integration of improved outpatient heart failure and valve care pathways.

A notable finding of our analysis is the high proportion of patients in advanced NYHA functional classes (III and IV) within the study cohort, representing a routine, unselected five-year series of TAVR cases treated at two high-volume German Heart Centers. While only 25.8% of patients were categorized in NYHA classes I and II, a striking 74.2% were classified in NYHA classes III and IV. This trend underscores the severity of the clinical condition in patients currently undergoing routine TAVR procedures. Given the ongoing demographic shift, characterized by an aging population and an increasing burden of comorbidities, the number of patients with advanced stages of HF requiring TAVR is expected to rise in the future. This observation not only highlights the growing complexity of TAVR indications but also underscores the need for more individualized management strategies to optimize outcomes in this high-risk cohort (23).

As the rising number of elderly patients with advanced HF presents a particular challenge during clinical decision making, in our cohort, patients classified as NYHA IV were significantly older, with a mean age of 80.5 ± 6.3 years, compared to those in the NYHA I/II and III classifications (I/II: 79.1 ± 5.9 years, III: 79.7 ± 6.4 years; *p* = 0.013). With advanced age, the prevalence of severe HF, right ventricular dysfunction, and frailty increases, potentially also reducing the ability to recover successfully after TAVR (24, 25). Thus, a significant proportion of patients in our cohort presented with moderate to severe tricuspid regurgitation, itself associated with increased mortality (20.4% in NYHA III, 26.7% in NYHA IV vs. 17.1% in NYHA I/II; *p* = 0.006). However, given the increasing life expectancy and the rising number of elderly patients, clinical decisions must place greater emphasis on the age-related comorbidity burden and focus on restoring the patient's quality of life, particularly in the context of TAVR, while also considering the comprehensive management of concomitant valvular disease, including mitral and tricuspid valve pathology (26).

Furthermore, our results demonstrate that patients in higher NYHA classes experienced significantly more peri-interventional complications. The need for prolonged vasopressor therapy (NYHA III: 5.5%, NYHA IV: 11.7% vs. NYHA I/II 1.6%), cardiopulmonary resuscitation (NYHA III: 2.2%, NYHA IV: 5.3% vs. NYHA I/II: 0.7%), and renal failure requiring RRT (NYHA III: 2.1%, NYHA IV: 6.8% vs. NYHA I/II: 0.2%), increased significantly with higher NYHA functional class (*p* **<** 0.001**)**. These complications, along with longer hospital stays, confirm that patients with advanced HF are at higher risk for immediate post-interventional challenges, presenting difficulties for healthcare professional teams and placing additional strain on healthcare resources and costs. By providing a detailed analysis of peri-interventional risk profiles across NYHA classes, our study extends existing literature by identifying patients with advanced functional impairment as a particularly vulnerable subgroup. While previous studies have established NYHA class as a prognostic marker, our findings indicate that patients in NYHA class IV represent a population with increased procedural vulnerability, underscoring the importance of careful patient selection, optimized timing of intervention, and individualized peri-procedural management.

Our data on long-term mortality finally confirm that the NYHA classification not only serves as an early risk parameter but also provides important prognostic value for long-term survival. 30-day mortality increased progressively with symptom severity, from 1.4% in NYHA I/II to 2.7% in NYHA III and 8.3% in NYHA IV (*p* < 0.001). Similarly, the NYHA classification demonstrated strong prognostic value for long-term outcomes, with 1-year mortality rising from 5.2% in patients with NYHA class I/II to 8.7% in class III and 19.2% in class IV (*p* < 0.001). These findings align with previous research, highlighting the impact of the overall symptom severity on post-TAVR outcomes (9, 27, 28). Moreover, this indicates that patients with advanced HF must be considered more carefully when selecting appropriate therapies to minimize the risk of poor outcomes.

Considering these findings, the role of the NYHA classification as a strong prognostic marker in pre-procedural assessment for TAVR becomes increasingly important. A timely and integrated assessment of clinical status, comorbidities, and NYHA classification may support informed treatment decision-making when differentiating between TAVR and alternative therapeutic strategies. Particularly in older patients or those with advanced HF, the potential risks and anticipated post-TAVR quality of life may be important considerations during clinical decision-making. A recent meta-analysis supports these findings and further demonstrates that urgent TAVR procedures in decompensated patients, compared to elective procedures, are associated with higher 30-day and in-hospital mortality, as well as increased vascular complications and acute renal failure (29). Future studies should investigate whether structured individualized pre-procedural stabilization strategies can improve outcomes in a growing high-risk population. Addressing both the clinical needs of an aging society and the economic challenges faced by healthcare systems, the focus should increasingly shift toward preemptive strategies that enable therapeutic interventions before patients reach advanced stages of heart failure. In this context, strengthened outpatient care structures, improved interdisciplinary networking, and the development of integrated heart failure and valve care pathways are essential to facilitate timely referral and continuous monitoring. Alongside these structural measures, efforts should be directed toward the identification of novel biomarkers and innovative targeted therapies, as well as improved diagnostic and referral strategies—such as advanced imaging techniques and personalized screening protocols—which may improve the early detection and longitudinal surveillance of aortic stenosis (30, 31). Results from recent studies confirm that early TAVR intervention, even in still asymptomatic patients, may significantly improve survival and quality of life while preventing the progression to HF and decompensation (12, 32).

## 5 Conclusions

Pre-procedural NYHA class provides important prognostic information in patients undergoing TAVR. Therefore, early intervention in cases of AS is essential to prevent clinical decompensation and to improve patient outcomes. However, in patients who present with higher NYHA classes, pre-procedural optimization strategies should be discussed. The incorporation of advanced diagnostic technologies and artificial intelligence offers promising opportunities for refined risk stratification and evidence-based decision-making. To minimize risk and increase procedural success, such individualized treatment strategies seem essential, particularly in an aging population with severe AS and concomitant HF. Given the increasing economic pressures on healthcare systems due to the growing number of elderly patients, optimizing patient management and procedural outcomes becomes even more critical to ensure the sustainability of healthcare resources.

### 5.1 Limitations

This study has several limitations. The retrospective design and the limited number of contributing centers may restrict the generalizability of the findings and do not allow for causal inference. Pre-procedural NYHA class was assigned by healthcare professionals, making it susceptible to individual interpretation and potential variability. NYHA functional class is a symptom-based classification and may be influenced by multiple concomitant cardiac and non-cardiac conditions. Objective measures of frailty or functional status were not included. Although CAD was systematically assessed and treated prior to TAVR, it was not feasible to attribute symptoms due to the retrospective design. Persistent dyspnea, as reflected by the NYHA functional class, may have been influenced by microvascular dysfunction, diffuse coronary disease, or advanced heart failure, despite angiographically adequate revascularization. Moreover, variations in procedural technique, device selection, and operator experience were not systematically captured. Important potential confounders, such as socioeconomic status, adherence to therapy, and access to follow-up care, were not assessed. Finally, clinical outcomes were not independently adjudicated, which may introduce reporting bias.

## Data Availability

The raw data supporting the conclusions of this article will be made available by the authors, without undue reservation.

## References

[1] Vahanian A Beyersdorf F Praz F Milojevic M Baldus S Bauersachs J 2021 ESC/EACTS guidelines for the management of valvular heart disease: developed by the task force for the management of valvular heart disease of the European society of cardiology (ESC) and the European association for cardio-thoracic surgery (EACTS). Eur Heart J. (2022) 43(7):561–632. 10.1093/eurheartj/ehab395 34453165

[2] Leon MB Smith CR Mack MJ Makkar RR Svensson LG Kodali SK Transcatheter or surgical aortic-valve replacement in intermediate-risk patients. N Engl J Med. (2016) 374(17):1609–20. 10.1056/NEJMoa1514616 27040324

[3] Mack MJ Leon MB Thourani VH Makkar R Kodali SK Russo M Transcatheter aortic-valve replacement with a balloon-expandable valve in low-risk patients. N Engl J Med. (2019) 380(18):1695–705. 10.1056/NEJMoa1814052 30883058

[4] Praz F Borger MA Lanz J Marin-Cuartas M Abreu A Adamo M 2025 ESC/EACTS guidelines for the management of valvular heart disease. Eur Heart J. (2025) 46(44):4635–736. 10.1093/eurheartj/ehaf194 40878295

[5] Fischer-Rasokat U Renker M Charitos EI Strunk C Treiber J Rolf A Cardiac decompensation of patients before transcatheter aortic valve implantation—clinical presentation, responsiveness to associated medication, and prognosis. Front Cardiovasc Med. (2023) 10:1232054. 10.3389/fcvm.2023.1232054 37942071 PMC10627789

[6] Chen S Redfors B Crowley A Ben-Yehuda O Summers M Hahn RT Impact of recent heart failure hospitalization on clinical outcomes in patients with severe aortic stenosis undergoing transcatheter aortic valve replacement: an analysis from the PARTNER 2 trial and registries. Eur J Heart Fail. (2020) 22(10):1866–74. 10.1002/ejhf.1841 32441856

[7] Strange JE Nouhravesh N Schou M Christensen DM Holt A Østergaard L High-risk admission prior to transcatheter aortic valve replacement and subsequent outcomes. Am Heart J. (2024) 268:53–60. 10.1016/j.ahj.2023.11.003 37972676

[8] McDonagh TA Metra M Adamo M Gardner RS Baumbach A Böhm M 2021 ESC guidelines for the diagnosis and treatment of acute and chronic heart failure: developed by the task force for the diagnosis and treatment of acute and chronic heart failure of the European Society of Cardiology (ESC) with the special contribution of the heart failure association (HFA) of the ESC. Rev Esp Cardiol (Engl Ed). (2022) 75(6):523. 10.1016/j.rec.2022.05.005 35636830

[9] Parikh PB Mack M Stone GW Anker SD Gilchrist IC Kalogeropoulos AP Transcatheter aortic valve replacement in heart failure. Eur J Heart Fail. (2024) 26(2):460–70. 10.1002/ejhf.3151 38297972

[10] Mengi S Januzzi JL Cavalcante JL Avvedimento M Galhardo A Bernier M Aortic stenosis, heart failure, and aortic valve replacement. JAMA Cardiol. (2024) 9(12):1159–68. 10.1001/jamacardio.2024.3486 39412797

[11] Coisne A Scotti A Latib A Montaigne D Ho EC Ludwig S Impact of moderate aortic stenosis on long-term clinical outcomes: a systematic review and meta-analysis. JACC Cardiovasc Interv. (2022) 15(16):1664–74. 10.1016/j.jcin.2022.06.022 35981841

[12] Généreux P Schwartz A Oldemeyer JB Pibarot P Cohen DJ Blanke P Transcatheter aortic-valve replacement for asymptomatic severe aortic stenosis. N Engl J Med. (2024) 392(3):217–27. 10.1056/NEJMoa2405880 39466903

[13] Mack MJ Leon MB Thourani VH Pibarot P Hahn RT Genereux P Transcatheter aortic-valve replacement in low-risk patients at five years. N Engl J Med. (2023) 389(21):1949–60. 10.1056/NEJMoa2307447 37874020

[14] Auffret V Bakhti A Leurent G Bedossa M Tomasi J Belhaj Soulami R Determinants and impact of heart failure readmission following transcatheter aortic valve replacement. Circ Cardiovasc Interv. (2020) 13(7):e008959. 10.1161/CIRCINTERVENTIONS.120.008959 32600108

[15] Durand E Doutriaux M Bettinger N Tron C Fauvel C Bauer F Incidence, prognostic impact, and predictive factors of readmission for heart failure after transcatheter aortic valve replacement. JACC Cardiovasc Interv. (2017) 10(23):2426–36. 10.1016/j.jcin.2017.09.010 29217006

[16] Leon MB Smith CR Mack M Miller DC Moses JW Svensson LG Transcatheter aortic-valve implantation for aortic stenosis in patients who cannot undergo surgery. N Engl J Med. (2010) 363(17):1597–607. 10.1056/NEJMoa1008232 20961243

[17] Adams DH Popma JJ Reardon MJ Yakubov SJ Coselli JS Deeb GM Transcatheter aortic-valve replacement with a self-expanding prosthesis. N Engl J Med. (2014) 370(19):1790–8. 10.1056/NEJMoa1400590 24678937

[18] Carroll JD Mack MJ Vemulapalli S Herrmann HC Gleason TG Hanzel G STS-ACC TVT registry of transcatheter aortic valve replacement. J Am Coll Cardiol. (2020) 76(21):2492–516. 10.1016/j.jacc.2020.09.595 33213729

[19] Gilard M Eltchaninoff H Donzeau-Gouge P Chevreul K Fajadet J Leprince P Late outcomes of transcatheter aortic valve replacement in high-risk patients: the FRANCE-2 registry. J Am Coll Cardiol. (2016) 68(15):1637–47. 10.1016/j.jacc.2016.07.747 27712776

[20] Taniguchi T Shirai S Ando K Arai Y Soga Y Hayashi M Impact of New York heart association functional class on outcomes after transcatheter aortic valve implantation. Cardiovasc Revasc Med. (2022) 38:19–26. 10.1016/j.carrev.2021.07.022 34340914

[21] Li S Tang BY Zhang B Wang C-P Yang S Chen J-B. Analysis of risk factors and establishment of a risk prediction model for cardiothoracic surgical intensive care unit readmission after heart valve surgery in China: a single-center study. Heart Lung. (2019) 48(1):61–8. 10.1016/j.hrtlng.2018.07.013 30149956

[22] Magruder JT Kashiouris M Grimm JC Duquaine D McGuinness B Russell S A predictive model and risk score for unplanned cardiac surgery intensive care unit readmissions. J Card Surg. (2015) 30(9):685–90. 10.1111/jocs.12589 26129715

[23] Bergmann T Sengupta PP Narula J. Is TAVR ready for the global aging population? Glob Heart. (2017) 12(4):291–9. 10.1016/j.gheart.2017.02.002 28433492

[24] Madanat L Allam M Khalili H Rabah A Tariq R Zamzam M Long-term survival and quality of life following transcatheter aortic valve replacement in nonagenarians. Am J Cardiol. (2024) 213:140–5. 10.1016/j.amjcard.2023.12.031 38134979

[25] Okoh AK Chauhan D Kang N Haik N Merlo A Cohen M The impact of frailty status on clinical and functional outcomes after transcatheter aortic valve replacement in nonagenarians with severe aortic stenosis. Catheter Cardiovasc Interv. (2017) 90(6):1000–6. 10.1002/ccd.27083 28463403

[26] Galatas C Afilalo J. Transcatheter aortic valve replacement over age 90: risks vs benefits. Clin Cardiol. (2020) 43(2):156–62. 10.1002/clc.23310 31840834 PMC7021650

[27] Adamo M Fiorina C Petronio AS Giannini C Tamburino C Barbanti M Comparison of early and long-term outcomes after transcatheter aortic valve implantation in patients with New York heart association functional class IV to those in class III and less. Am J Cardiol. (2018) 122(10):1718–26. 10.1016/j.amjcard.2018.08.006 30227961

[28] Kim CA Rasania SP Afilalo J Popma JJ Lipsitz LA Kim DH. Functional status and quality of life after transcatheter aortic valve replacement: a systematic review. Ann Intern Med. (2014) 160(4):243–54. 10.7326/M13-1316 24727842 PMC4039034

[29] Apostolos A Ktenopoulos N Chlorogiannis DD Katsaros O Konstantinou K Drakopoulou M Mortality rates in patients undergoing urgent versus elective transcatheter aortic valve replacement: a meta-analysis. Angiology. (2024) 76:784–95. 10.1177/00033197241245733 38613209

[30] Zhang Y Wang M Zhang E Wu Y. Artificial intelligence in the screening, diagnosis, and management of aortic stenosis. Rev Cardiovasc Med. (2024) 25(1):31. 10.31083/j.rcm2501031 39077660 PMC11262349

[31] Sun S Yeh L Imanzadeh A Kooraki S Kheradvar A Bedayat A. The current landscape of artificial intelligence in imaging for transcatheter aortic valve replacement. Curr Radiol Rep. (2024) 12(11-12):113–20. 10.1007/s40134-024-00431-w 39483792 PMC11526784

[32] Beerkens FJ Tang GHL Kini AS Lerakis S Dangas GD Mehran R Transcatheter aortic valve replacement beyond severe aortic stenosis: JACC state-of-the-art review. J Am Coll Cardiol. (2025) 85(9):944–64. 10.1016/j.jacc.2024.11.051 40044299

